# Effective but fragile? Responses to repeated nudge-based messages for preventing the spread of COVID-19 infection

**DOI:** 10.1007/s42973-021-00076-w

**Published:** 2021-06-14

**Authors:** Shusaku Sasaki, Hirofumi Kurokawa, Fumio Ohtake

**Affiliations:** 1 Faculty of Economics, Tohoku Gakuin University, 1-3-1 Tsuchitoi, Aoba-ku, Sendai, Miyagi 985-8537 Japan; 2 School of Economics and Management, University of Hyogo, 8-2-1 Gakuennishi-machi Nishi-ku, Kobe City, Hyogo 651-2197 Japan; 3 Center for Infectious Disease Education and Research (CiDER), Osaka University, 2-8, Yamadaoka, Suita City, Osaka 565-0871 Japan

**Keywords:** Infectious diseases, COVID-19, Nudge, Behavioral economics, Altruism, Panel survey experiment

## Abstract

Nudge-based messages have been employed in various countries to encourage voluntary contact-avoidance and infection-prevention behaviors to control the spread of COVID-19. People have been repeatedly exposed to such messages; however, whether the messages keep exerting a significant impact over time remains unclear. From April to August 2020, we conducted a four-wave online survey experiment to examine how five types of nudge-based messages influence Japanese people’s self-reported preventive behaviors. In particular, we investigate how their behaviors are affected by repeated displays over time. The analysis with 4241 participants finds that only a gain-framed altruistic message, emphasizing their behavioral adherence would protect the lives of people close to them, reduces their frequency of going out and contacting others. We do not find similar behavioral changes in messages that contain an altruistic element but emphasize it in a loss-frame or describe their behavioral adherence as protecting both one’s own and others’ lives. Furthermore, the behavioral change effect of the gain-framed altruistic message disappears in the third and fourth waves, although its impact of reinforcing intentions remains. This message has even an adverse effect of worsening the compliance level of infection-prevention behaviors for the subgroup who went out less frequently before the experiment. The study’s results imply that when using nudge-based messages as a countermeasure for COVID-19, policymakers and practitioners need to carefully scrutinize the message elements and wording and examine to whom and how the messages should be delivered while considering their potential adverse and side effects.

## Introduction

Since December 2019, the COVID-19 outbreak has posed a global health threat. Until effective vaccination is completed for a sufficient number of people and herd immunity is acquired, controlling the spread of the infection is crucial. Staying home, reducing contact with other people, practicing social distancing, frequent and proper handwashing, disinfecting hands, and wearing a mask have been reported to be effective strategies for preventing the spread of the infection (World Health Organization, 2020). Hence, it is essential to ascertain which types of intervention encourage individuals to take preventive measures.

Urban lockdowns have been enforced in most countries, restricting individuals from going out and operating businesses with potential penalties. Governments aim to reduce social contact and visits to places with a high risk of infection. However, some countries, including Japan, have been unable to enforce compulsory interventions under their current laws. Such countries have implemented non-compulsory interventions (e.g., requests/guidance), urging people to voluntarily take preventive measures. Others also have lifted previously implemented lockdowns and switched to non-compulsory interventions to encourage voluntary preventive behaviors, simultaneously resuming economic activity. Therefore, understanding which non-compulsory interventions effectively promote voluntary preventive behaviors is crucial.

In the field of behavioral economics, “nudge” is generally known as a method to guide people’s behavior toward a socially desirable direction while maintaining their freedom of choice (Thaler & Sunstein, 2009). It has been reported that nudge-based interventions can promote positive behaviors in the medical and health fields (Patel et al., 2018; Vallgårda, 2012).

In the COVID-19 pandemic, policymakers and medical practitioners have developed messages that contain elements and wording based on nudges and use nudge-based messages to encourage people to take preventive measures. Through social media and television broadcasting, altruistic messages have been spread, emphasizing that taking such steps will save the lives of others (British Broadcasting Corporation News, 2020; New Zealand Police, 2020).^1^

An increasing number of studies have experimentally evaluated the impact of nudge-based messages on people’s perceptions and intentions to prevent the spread of COVID-19. For example, Lunn et al. (2020) find that messages emphasizing that people could cause the exponential spread of the infection are more effective in fostering the intention to take preventive measures than those that instruct them to maintain a social distance of two meters. Utych and Fowler (2020) show that people residing in an outbreak region are likely to recognize the risks of COVID-19 when exposed to a message emphasizing how COVID-19 poses a danger to both elderly and young people. Luttrell and Petty (2020) find that people consider messages that focus on others as more persuasive than self-focused messages, especially those who perceive public health as a moral issue.

As mentioned above, some studies have found that altruistic messages focusing on others have the desired impact on people’s perceptions and intentions for infection-prevention. However, Jordan et al. (2020) report that self-interested, public-interested, and self- and public-interested messages are similarly effective. Barari et al. (2020) find that nudge-based messages do not have an additional stimulatory effect on those who are already complying with preventive instruction. Favero and Pedersen (2020) also find no promoting effect of such messages. Thus, the results are mixed.

When setting behaviors as the dependent variable rather than intentions, very few studies have demonstrated the effectiveness of nudge-based messages. Falco and Zaccagni (2020) find that a message that emphasizes taking preventive measures benefits “you and your family” increases the intention to engage in such actions while having no observable effect on behaviors. Barari et al. (2020) and Everett et al. (2020) also find no behavioral change following such messages. However, Moriwaki et al. (2020) use information on smartphones’ GPS and Japanese users’ spatial movements and show that nudge-based messages promote their avoidance of closed spaces, crowded spaces, and close contact during weekends. Krpan et al. (2021) use self-reported behaviors and show the possibility that the nudge for information provision promotes contact avoidance in those who recently embraced avoidance, while such an intervention has the backfire effect of reducing the frequency of avoidance in those who previously avoided contact.

This study adds to the literature another evidence on the effects of nudge-based messages on people’s adherence to behavioral measures to control the spread of COVID-19. In particular, we conduct a four-wave online survey experiment on residents in Japan and examine how the impact of nudge-based messages is affected by multiple exposures. To the best of our knowledge, no other study has examined the effects of repeatedly displaying messages for the same individual in this context; therefore, this study provides unique insights. The COVID-19 pandemic has already lasted for over a year, and governments have repeatedly used messages calling for contact-avoidance and infection-prevention. Therefore, whether the same messages will continue to be effective is crucial from a policy perspective.

The survey experiment comprises four waves over three months, from the end of April to the beginning of August 2020. In the first wave, we present nudge-based messages as a randomized controlled trial and ask survey participants about their intentions to refrain from going out, avoid contact with others, and prevent infection. In the second wave, we ascertain the extent to which they adhere to those behaviors since the first wave and present the same nudge-based message and again ask for participants’ intentions. For the third and fourth waves, we repeat the same structure as the second wave.

This study uses five nudge-based messages: (1) Gain-framed altruistic message: “By avoiding contact with others and taking measures to prevent infection, you can protect the lives of people close to you;” (2) Loss-framed altruistic message: “If you do not take such measures, you will expose people close to you to danger;” (3) Selfish message: “By taking such measures, you can protect your own life;” (4) Altruistic and selfish message: “By taking such measures, you can protect your own life and the lives of people close to you;” and (5) Simple message: “Stay home.”

These nudge-based messages have three primary characteristics. First, we compare the message that emphasizes the interests of others (message 1) with the one that emphasizes self-interest (message 3). Previous behavioral economics studies have found that many people act not only in their interest but also in the interest of others, as in the case of charitable giving (Andreoni, 1990) and other cooperative behaviors (Fehr & Gachter, 2000). Recent studies have suggested that people’s prosociality is related to their preventive behavior during the COVID-19 pandemic (Campos-Mercade et al., 2021; Müller & Rau, 2021). The selfish message that one can self-protect from infection through contact-avoidance and infection-prevention behavior promotes compliance in those who do not want to be infected due to selfish motives. However, it does not promote avoidance behavior in those who are not afraid of being infected. An altruistic message that explains how to avoid infecting others through one’s actions and emphasizes prosocial motives can draw the attention of such individuals to the benefit of others, making them more willing to engage in contact-avoidance and infection-prevention measures. For instance, some studies have shown that messages emphasizing social interests enhance people’s willingness to receive influenza vaccines (Betsch et al., 2017).

Message (4) emphasizes both sets of interests. Since this message should reach both those who care about their own interests and those who focus on the interests of others, we expect it to strengthen people’s intentions to avoid contagion and promote their infection-prevention behavior. However, simultaneously promoting both sets of interests may “crowd out” the altruistic motivation in those attempting to behave in the interests of others, thus impeding their actions (Gneezy & Rustichini, 2000).

Second, we compare message (1), emphasizing the interests of others by framing it in terms of “gain,” and message (2), framed in terms of “loss.” The prospect theory of Tversky and Kahneman (1981) states that people’s choices depend on whether they are framed in terms of “gain” or “loss,” even if their substance is essentially equivalent. A loss-framed message is expected to be more effective for behavioral changes than a gain-framed message. However, in practice, which frame is more effective seems to depend on the context. For example, in the health and medical fields, “gain frames” are particularly effective in promoting behaviors aimed at preventing the onset of diseases, including the use of sunscreen (Detweiler et al., 1999) and quitting smoking (Toll et al., 2007). In contrast, for promoting disease-discovery behaviors, including mammography tests (Schneider et al., 2001), “loss” frames are more effective (Rothman et al., 2006). Therefore, in the COVID-19 pandemic context, the gain-framed message may be more effective in promoting contact-avoidance and infection-prevention behavior.

Third, we set as another reference message (5), which simply urges a specific action. It is generally known that simple expressions are easier to understand (Behavioural Insights Team, 2014; Sunstein, 2014). The simplification of actionable instructions has long been the subject of debate in public health (Zarcadoolas, 2011). The message “Stay Home” has also been used through social media and online campaigns worldwide.

The remainder of this paper is organized as follows: Sect. 2 describes the study’s experimental design. Section 3 reports the primary results, and Sect. 4 details the supplementary results. Section 5 discusses the findings and their practical implications and concludes, noting the study’s limitations.

## Experimental design

### Overview of experiment

We conducted a four-wave survey experiment in the following periods: April 28–April 30 (the first wave), May 8–May 13 (the second wave), June 8–June 12 (the third wave), and July 28–August 3 (the fourth wave). We commissioned MyVoiceCom Co., Ltd., a company engaged in online surveys, to conduct the survey experiment. We extracted participants throughout Japan using their monitors, such that the individuals’ gender and age (20–69 years) ratios would be equal. We addressed 4241 individuals who joined all four waves and provided valid answers.^2^ Table 7 in “Appendix” shows the response rates in the second, third, and fourth survey waves for each group, setting the number of valid responses in the first wave as the denominator. The response rates decrease with each wave, with no difference observed between groups. The response rates in the fourth and final wave are approximately 80% regardless of the group.

**Fig. 1:**
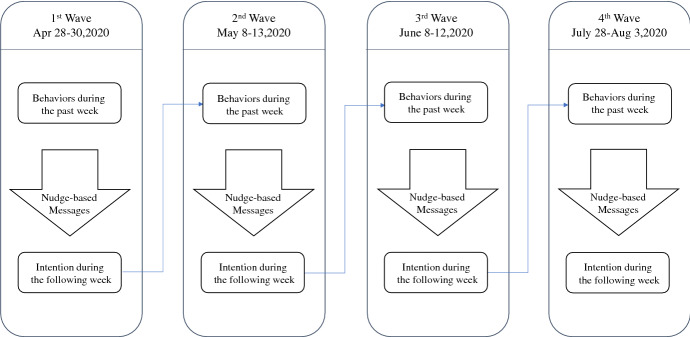
Survey structures

Figure 1 details the structure of each survey wave. The first wave captures participants’ attributes, including gender, age, address, the frequency of contact with others (by going out), and the frequency of taking behavioral measures to prevent infection in the week before the survey. Next, we randomly assign participants to one of the control groups and five nudge-based message groups and expose each group to a different message. We then ask participants their intentions to take contact-avoidance and infection-prevention measures during the following week. Finally, we ask them questions about their socio-economic attributes, including their educational background, family structure, and household income.

The second, third, and fourth survey waves have the same structure. In these waves, we present questions about the frequency of contact with others (by going out) and their infection-prevention measures in the week before each survey wave. We then expose them to the same nudge-based messages as in the first wave, and ask them questions to determine their intentions to refrain from going out, avoiding contact with others, and preventing infection.

**Fig. 2:**
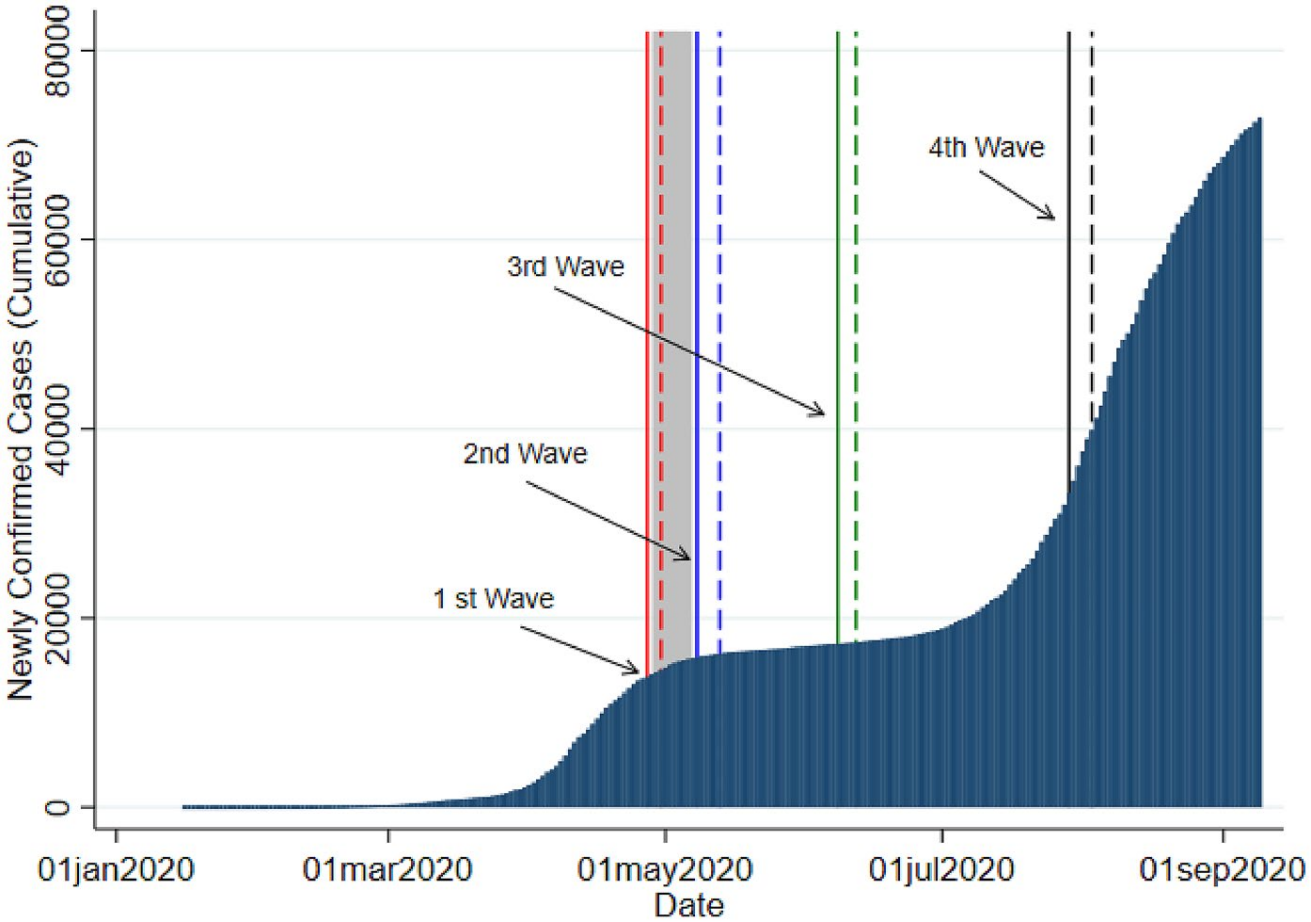
Cumulative number of confirmed new infections in Japan. *Notes* The data on newly confirmed case are taken from the Ministry of Health, Labour, and Welfare (https://www.mhlw.go.jp/stf/covid-19/open-data.html). A solid (dash) line shows the start (end) point of each wave. Gray shade (April 29-May 6) shows “Golden Week” (GW), containing several holidays in close succession. The Japanese government declared a state of emergency in seven prefectures (Tokyo, Saitama, Chiba, Kanagawa, Osaka, Hyogo, and Fukuoka) on April 7 and the other prefectures on April 16. The government removed the state of emergency in 39 prefectures on May 14 and 3 prefectures (Kyoto, Osaka, and Hyogo) on May 21 and the other prefectures (Tokyo, Saitama, Chiba, Kanagawa, Hokkaido) on May 25

Figure 2 displays the cumulative number of confirmed new infections in Japan vis á vis the schedule of the survey experiment. The first and second waves were carried out when the Japanese government declared a state of emergency (“kinkyu-jitai-sengen” in Japanese). Japan has primarily relied on non-compulsory interventions to control the spread of the infection, including different levels of requests and guidance. Declarations allow local governments to request restaurants and other stores to shorten their business hours and pay cooperation money to stores that adhere to the request. Following the government’s declarations, the level of awareness regarding the spread of COVID-19 infection climaxed among the Japanese people. The period between the first and second waves is referred to as the “Golden Week” in Japan. It is a holiday period during which many people usually travel to national tourist spots. Amid the pandemic, it was extremely important to control people’s movements, especially during the holiday week, promoting avoidance of close contact and taking thorough measures to prevent further spread of the infection.

Thus, the first part of this experiment examines the effect of the nudge-based messages displayed in the first survey wave on participants’ contact-avoidance and infection-prevention behaviors during the Golden Week. In other words, we examine the short-term effects of the messages.

The third and fourth waves were carried out when the number of confirmed new infections began settling down, and the government removed the state of emergency. The level of awareness regarding the spread of COVID-19 started to decline.^3^ In addition, more than 1 month passed between the second and the third wave and between the third and the fourth wave.

Thus, the second part of the experiment examines the effect of repeatedly displaying nudge-based messages on participants’ contact-avoidance and infection-prevention behaviors during the lull of the COVID-19 infection. Hence, we examine the relatively long-term effects of such messages.

For this study, we obtained ex-ante approval from the ethics committee of Graduate School of Economics, Osaka University.

### Nudge-based messages

We first provide participants with general information about COVID-19 (the declaration of emergency, mortality rate, and transmission routes, among others) to all groups (see the actual screen in Fig. 4 of the “Appendix”). We then introduce three effective measures to prevent the infection.^4^

*“To prevent infection,*

- *Reducing contact with others;*
- *Avoiding the “3 Cs” of closed spaces, crowded spaces, and close contact;*
- *Practicing proper hand washing and wearing a mask*

*are effective.”*

In the intervention groups, one of the five nudge-based messages is randomly displayed, in addition to the above introduction. The specific messages read as follows (see the actual screens in Figs. 5, 6, 7, 8, 9, 10 of the “Appendix”):

1. *Gain-framed altruistic message:*

*By refraining from going out, avoiding the “3 Cs,” washing your hands, and wearing a mask, you can protect the lives of people close to you.*

2. *Loss-framed altruistic message:*

*By going out, not avoiding the “3 Cs,” and not washing your hands or wearing a mask, you will put the lives of people close to you at risk.*

3. *Selfish message:*

*By refraining from going out, avoiding the “3 Cs,” washing your hands, and wearing a mask, you can protect your own life.*

4. *Altruistic and selfish message:*

*By refraining from going out, avoiding the “3 Cs,” washing your hands, and wearing a mask, you can protect the lives of yourself and people close to you.*

5. *Simple message:*

*Stay home. You can protect the lives of people close to you.*

While the contents of the nudge-based messages are essentially the same across the survey waves, we fine-tune the message wording to reflect changes in the social situation. At the time of the first and second survey waves, the Japanese government declared a state of emergency and asked people to refrain from leaving their houses, avoid social contact, and prevent infection. Thus, we use the phrase “reducing contact with others” in the common message in the first part of the experiment. At the time of the third and fourth waves, the government was gradually focusing on preventing the spread of the infection and reopening economic activities. Thus, in the second part of the experiment, we use the wording “keeping space between you and others.”

### Outcome measures

This study’s primary outcome variable is the “contact-avoidance behavior INDEX.” In each wave, we measure the frequency of the following 10 contact behaviors in the week before the survey (from “0: never” to “7: almost every day”).^5^

1. *Go to a tavern or bar;*
2. *Go to a restaurant;*
3. *Go to a cafe;*
4. *Go to a supermarket or grocery store;*
5. *Go to a gym;*
6. *Go to work;*
7. *Travel on public transportation such as trains and buses;*
8. *Travel by plane;*
9. *Participate in small gatherings and events (excluding online events);*
10. *Participate in large gatherings and events (excluding online events).*

We invert the participants’ responses to the 10 behavioral measures and convert them into measures of contact avoidance. We add these measures and divide the total value by 10. We call this value the “contact-avoidance behavior INDEX.” If we set each item as the dependent variable and perform regression analysis respectively, some intervention effects may be spuriously significant. To address this concern to some degree, we use a comprehensive index.^6^

**Fig. 3:**
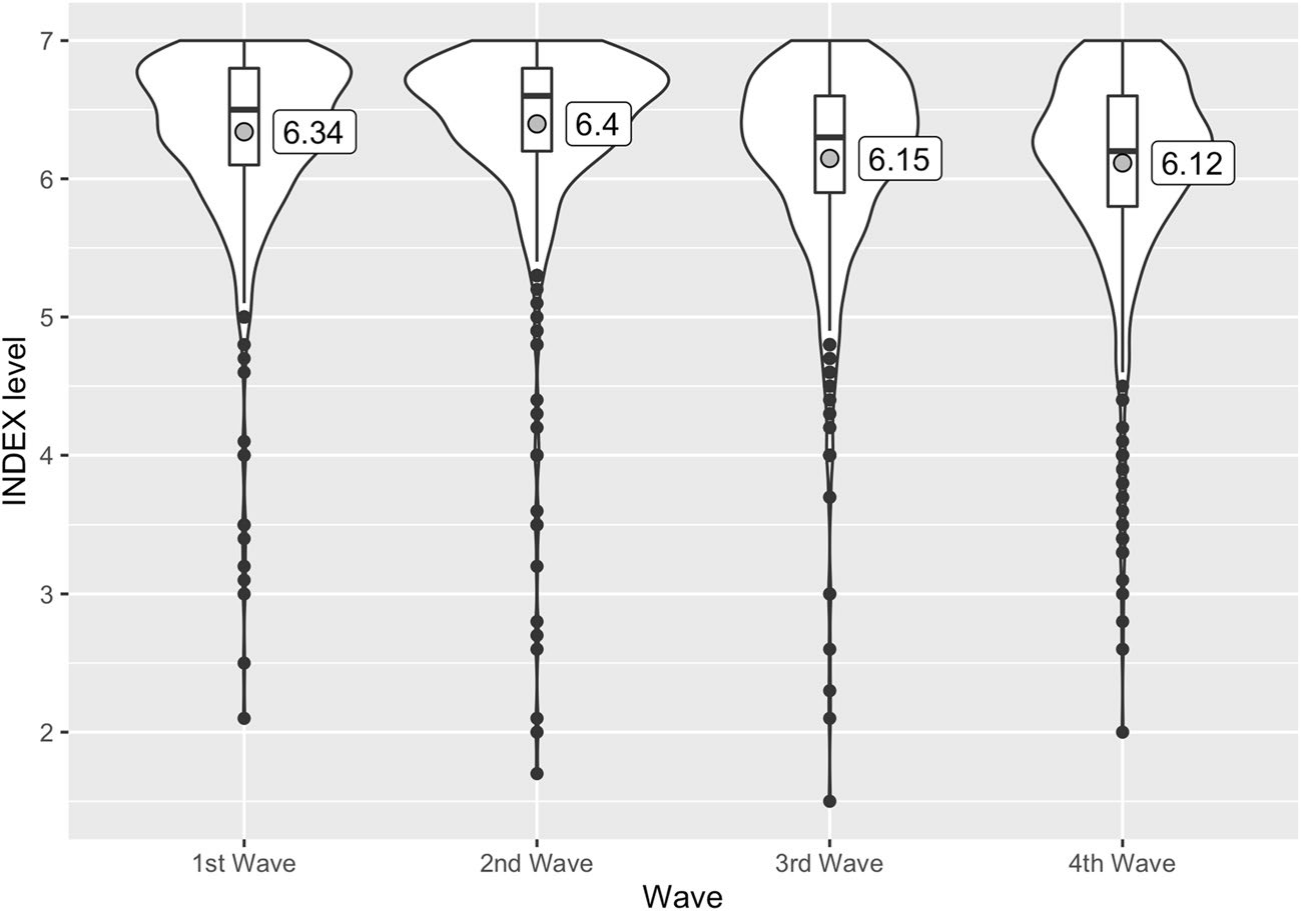
Contact-avoidance behavior INDEX for the control group in each wave. *Notes* The maximum value of this index is 7, which refers to the state wherein contact avoidance is most thorough. In contrast, the minimum value is 0, which refers to the state wherein contact avoidance is least thorough

**Fig. 4:**
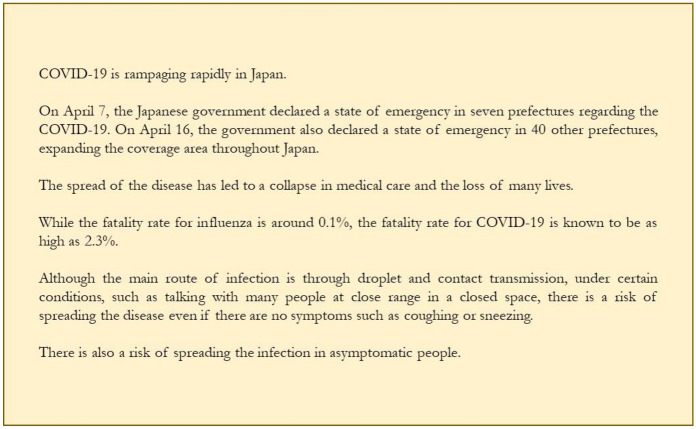
Screen of general information on COVID-19

**Fig. 5:**
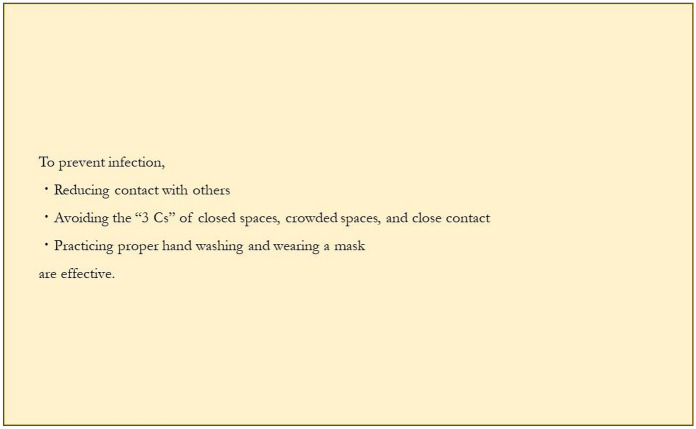
**Screen of control group**

**Fig. 6:**
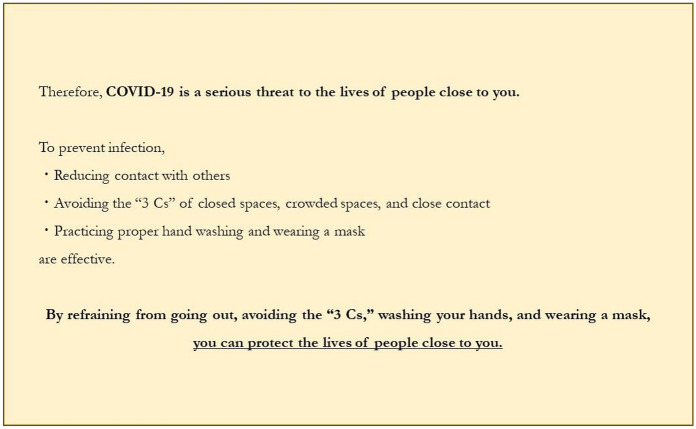
Screen of “Gain-framed altruistic message” group

**Fig. 7:**
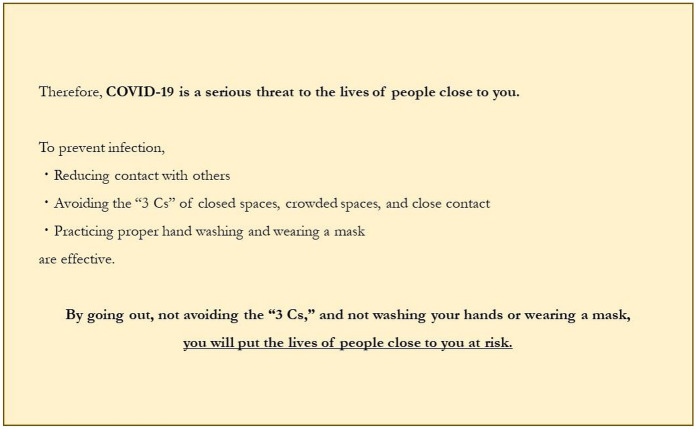
Screen of “Loss-framed altruistic message” group

**Fig. 8:**
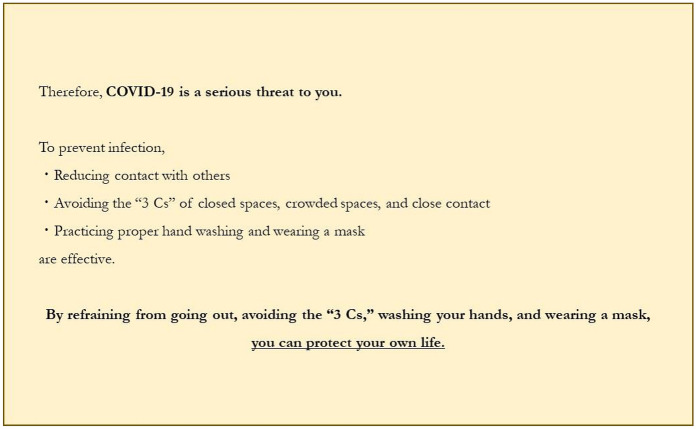
Screen of “Selfish message” group

**Fig. 9:**
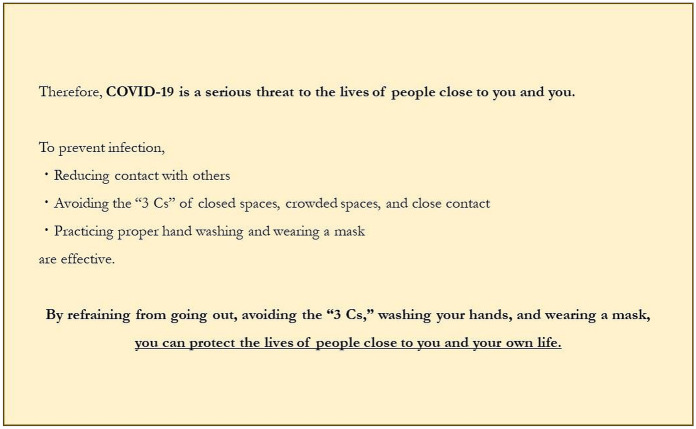
Screen of “Altruistic and selfish message” group

**Fig. 10:**
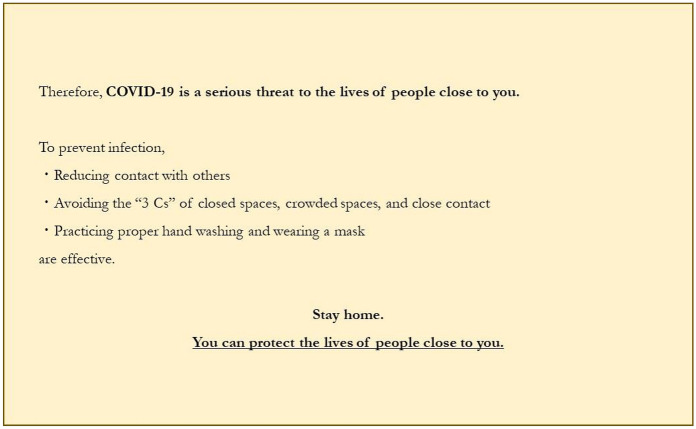
Screen of “Simple message” group

Figure 3 shows the average index for the control group, which is not exposed to any nudge-based messages, for each survey wave. The first and second waves show relatively high levels of the index, while low index levels characterize the third and fourth waves. As mentioned earlier, the Japanese government declared a state of emergency during the first part of the experiment and removed it during the second part. The results for the control group indicate that the level of contact avoidance was quite high during the state of emergency but slightly decreased after its removal.

The second outcome variable is the “infection-prevention behavior INDEX.” In each survey wave, we measure the frequency of 15 infection-prevention behaviors in the week before the survey (from “0: never” to “7: almost every day”), as shown below.^7^ This variable is designed to capture the frequency of people taking measures to reduce the risk of infection, assuming that they have some opportunities to go out and have contact with others. We add these measures and divide the total value by 15, calling this value the “infection-prevention behavior INDEX.”

1. *When you need to cough or sneeze, place a mask or handkerchief over your mouth (“cough etiquette”);*
2. *Practice gargling and frequent hand washing and disinfection of hands and fingers with alcohol;*
3. *Try to avoid touching your face;*
4. *Always wear a mask when talking;*
5. *Avoid shaking hands;*
6. *Talk with others *via* phone or video call whenever possible;*
7. *Designate one person to shop or go out in a small group and during times when stores are not crowded;*
8. *Use cashless payment methods (credit cards, electronic money, *etc*.) instead of cash;*
9. *Use take-out or home delivery services instead of going to restaurants;*
10. *Use delivery or mail-order services for larger purchases;*
11. *Always wear a mask when going out;*
12. *Avoid going out if you feel unwell;*
13. *Try to stay home as much as possible even if you are not ill;*
14. *Get plenty of rest and sleep;*
15. *Eat nutritious foods.*

We create the 10 and 15 behavioral measures based on the studies by Jordan et al. (2020) and Muto et al. (2020) and fine-tune them based on the Japanese context.

While the two outcome variables above reflect participants’ self-reported behaviors, the third outcome variable indicates prior intentions. In particular, we will show the results of the intention to comply with contact-avoidance behavior, which is our primary outcome variable. In each wave, we present the same behavioral measures immediately after the display of the nudge-based messages. We then ascertain the intention to adhere to those measures for the following week (from “0: no intention of stopping” to “7: will definitely stop”).

### Descriptive statistics

Table 1 shows the descriptive statistics of the socio-economic attributes of the analysis sample for each group. From test results, we confirm that there are no differences in age, gender, educational years, married status, and annual household income information between the control and intervention groups. We also confirm that the levels of the contact-avoidance INDEX and the infection-prevention behavior INDEX are balanced among the groups, overall.

**Table 1:** Descriptive statistics by group

*Variable name:*		Control	Gain-framed altruistic	Loss-framed altruistic	Selfish	Selfish and altruistic	Simple
	*N* =	704	717	697	725	684	714
Age	Mean	46.73	46.29	46.68	45.96	46.70	46.45
	S.D	[13.62]	[13.53]	[13.48]	[13.76]	[13.87]	[13.92]
Female dummy	Mean	0.49	0.50	0.50	0.50	0.52	0.49
	S.D	[0.50]	[0.50]	[0.50]	[0.50]	[0.50]	[0.50]
Educational year	Mean	14.58	14.60	14.67	14.63	14.66	14.73
	S.D	[2.09]	[2.05]	[1.97]	[2.03]	[2.06]	[2.06]
Married dummy	Mean	0.57	0.59	0.60	0.56	0.57	0.55
	S.D	[0.49]	[0.49]	[0.49]	[0.50]	[0.49]	[0.50]
Annual household income (Unit: 10 thousand yen)	Mean	606.16	622.92	611.07	631.46	627.84	628.98
	S.D	[344.90]	[378.02]	[359.44]	[373.06]	[343.79]	[376.24]
No income information dummy	Mean	0.19	0.16	0.19	0.19	0.19	0.17
	S.D	[0.39]	[0.37]	[0.39]	[0.39]	[0.39]	[0.38]

*Note* Some participants did not answer annual household income. We imputed the average amount of income for such respondents while considering that they did not answer it by using the variable of no income information

Next, we compare the analysis sample of those who provided valid responses in all four survey waves with those who did not, and examine their differences. Table 8 in “Appendix” shows that some systematic differences exist in their socio-economic attributes, while such differences occur in the same way for each group. Specifically, in all groups, the age, proportion of males, and annual household income in the analysis sample are higher than those of the other sample. Some of these differences are found to be statistically significant. Survey monitors with these characteristics may be more likely to participate in surveys continuously. According to the survey company of MyVoiceCom Co., Ltd., younger monitors are likely to drop out of online surveys. Hence, the study’s results may not fully capture the impact of nudge-based messages on younger generations.

In contrast, no notable differences are observed in psychological characteristics between the analysis and the remainder of the sample, including altruism and general trust. Since this study examines the effects of messages characterized by an altruistic element, we are concerned that a large number of altruistic samples might remain in the continuous waves, and thus their effects might be biased upward. However, we confirm that this is not a concern in this study.

## Empirical strategy and results

We evaluate the effects of the above five nudge-based messages on contact-avoidance and infection-prevention behaviors using a fixed effects estimation and the following model specification (1):

1 $$y_{i,t} = \alpha + \beta W + \gamma_{1} W_{t} \times N1_{i} + \gamma_{2} W_{t} \times N2_{i} + \gamma_{3} W_{t} \times N3_{i} + \gamma_{4} W_{t} \times N4_{i} + \gamma_{5} W_{t} \times N5_{i} + u_{i,t} .$$

Our estimations use two periods of data: the first and second waves, the first and third waves, and the first and fourth waves, respectively.^8^ The dependent variable ${y}_{i,t}$ refers to participant $i$’s contact-avoidance behavior INDEX or infection-prevention behavior INDEX in wave $t$. $\alpha$ is a constant term that represents the level of the index in the first wave. ${W}_{t}$ is a dummy variable that identifies the second, third, or fourth wave, and its parameter $\beta$ represents the degree to which the level of the index has changed from that of the first wave. $N1-N5$ are dummy variables that identify groups exposed to five different nudge-based messages; ${\gamma }_{1}-{\gamma }_{5}$ are the treatment effects and represent the extent to which the level of the index changes from the first to the second, third, or fourth wave as a result of viewing each nudge-based message. Finally, ${u}_{i,t}$ is an error term that consists of ${\eta }_{i}$ (time-invariant individual effect) and ${\nu }_{i,t}$ (a random error term).

First, we conduct the estimation using all the analysis samples. Second, we employ the contact-avoidance behavior INDEX in the first wave and divide the analysis sample into two subsamples: those who were relatively compliant with contact-avoidance instructions before the experiment ($\mathrm{INDEX}\ge 6.5$, *N* = 2128) and those who were not ($\mathrm{INDEX}<6.5$, *N* = 2113). Then, we estimate the proposed regression model for each subsample.^9^

### Contact-avoidance behavior INDEX

We report the results obtained when using the contact-avoidance behavior INDEX as the dependent variable. Our first finding is that the gain-framed altruistic message further promotes contact-avoidance behavior between the first and the second wave. This effect is observed in those who were less compliant with contact-avoidance recommendations and went out more frequently before the experiment. These individuals seem to decrease their frequency of going out to cafes and using public transportation. However, the effect is short-term and disappears in the third and fourth waves. The second finding is that the other four types of nudge-based messages have no promoting effect on the contact-avoidance behavior in either the short or long term. In the long run, the selfish message may even have the opposite effect of making participants neglect contact avoidance compared to the control group.

Column 1 of Table 2 shows the results obtained when using the data of the first and second waves. This step of the analysis captures how nudge-based messages affect the change in contact-avoidance behavior from the first to the second wave under the state of emergency. First, the coefficient on the dummy variable for the second wave is 0.057 (*p* < 0.05). This result implies that the control group participants (not exposed to nudge-based messages) increase their level of contact-avoidance behavior INDEX from the first to the second wave. Second, among the five treatment groups (all exposed to nudge-based messages), the coefficient on the gain-framed altruistic message is 0.071 and statistically significant at the 5% level, implying that the level of contact-avoidance behavior INDEX further increases among those exposed to this altruistic message. The other four messages have no statistically significant effect.

**Table 2:** Message effects on contact-avoidance behavior INDEX

*Method:* fixed effects estimation	(1)	(2)	(3)	(4)	(5)	(6)	(7)	(8)	(9)
*Dependent variable:*	Full-sample			Sub-sample A: Those who were previously more compliant with contact-avoidance instructions			Sub-sample B: Those who were previously less compliant with contact-avoidance instructions		
Contact-avoidance behavior INDEX									
Wave2	0.057**			− 0.097***			0.214***		
	(0.024)			(0.026)			(0.039)		
Wave2 × Gain-framed altruistic	0.071**			0.007			0.133**		
	(0.036)			(0.034)			(0.060)		
Wave2 × Loss-framed altruistic	0.029			0.006			0.053		
	(0.033)			(0.034)			(0.053)		
Wave2 × Selfish	0.012			0.039			− 0.025		
	(0.031)			(0.033)			(0.051)		
Wave2 × Selfish and altruistic	0.033			0.054*			0.009		
	(0.033)			(0.029)			(0.057)		
Wave2 × Simple	− 0.008			− 0.011			0.002		
	(0.035)			(0.039)			(0.056)		
Wave3		− 0.193***			− 0.282***			− 0.102***	
		(0.023)			(0.024)			(0.039)	
Wave3 × Gain-framed altruistic		0.036			0.014			0.057	
		(0.036)			(0.038)			(0.061)	
Wave3 × Loss-framed altruistic		0.015			− 0.016			0.048	
		(0.033)			(0.034)			(0.055)	
Wave3 × Selfish		− 0.058*			− 0.031			− 0.091	
		(0.035)			(0.039)			(0.057)	
Wave3 × Selfish and altruistic		0.030			− 0.005			0.064	
		(0.034)			(0.039)			(0.056)	
Wave3 × Simple		0.016			− 0.001			0.038	
		(0.036)			(0.041)			(0.059)	
Wave4			− 0.225***			− 0.346***			− 0.102**
			(0.025)			(0.027)			(0.042)
Wave4 × Gain-framed altruistic			0.012			− 0.006			0.028
			(0.038)			(0.040)			(0.064)
Wave4 × Loss-framed altruistic			− 0.014			− 0.020			− 0.008
			(0.037)			(0.043)			(0.058)
Wave4 × Selfish			− 0.072*			− 0.041			− 0.110*
			(0.037)			(0.043)			(0.059)
Wave4 × Selfish and altruistic			0.009			0.004			0.011
			(0.037)			(0.041)			(0.061)
Wave4 × Simple			− 0.001			− 0.035			0.041
			(0.037)			(0.042)			(0.059)
Constant term	6.315***	6.315***	6.315***	6.751***	6.751***	6.751***	5.877***	5.877***	5.877***
	(0.005)	(0.005)	(0.006)	(0.005)	(0.006)	(0.006)	(0.008)	(0.009)	(0.009)
Number of observations	8482	8482	8482	4256	4256	4256	4226	4226	4226
Number of participants	4241	4241	4241	2128	2128	2128	2113	2113	2113

*Notes* Cluster robust standard errors at individual level are in parentheses; ***p < 0.01, **p < 0.05, *p < 0.1. We employ the contact-avoidance behavior INDEX in the first wave and divide the analysis sample into two subsamples: those who were relatively compliant with contact-avoidance instructions before the experiment (Sub-sample A: INDEX ≥ 6.5, *N* = 2128) and those who were not (Sub-sample B: INDEX < 6.5, *N* = 2113)

Columns 2 and 3 show the results obtained when using the data of the first and third waves and the first and fourth waves, respectively. These findings describe how multiple nudge-based message exposures (two or three times) affect contact-avoidance behaviors. Since more than 1 month passes between the message exposure and the ascertainment of the level of the contact-avoidance behavior INDEX, we capture the relatively long-term effects of multiple message exposures. First, the coefficients on each wave are negative and equal to − 0.193 and − 0.225, respectively (*p* < 0.01), implying that control group participants decrease the level of the contact-avoidance behavior INDEX from the first wave, under the state of emergency, to the third or fourth wave, when it was removed. Next, the gain-framed altruistic message's promoting effect is not observed in the third and fourth waves. However, the effect of the selfish message is − 0.058 in the third wave and − 0.072 in the fourth wave. These results imply that in addition to the overall decrease in the level of contact-avoidance behavior INDEX during this period, those exposed to the selfish message further decrease their compliance level. The statistical significance of this effect is 10%; however, it is still observed after more than 1 month since the most recent message exposure. Therefore, this effect may be persistent.

We divide the analysis sample into two subsamples: those who were relatively compliant with contact avoidance at the time before the experiment and those who were not. Then, we conduct the estimation for each subsample. Columns 4–6 show the results for the first subsample and columns 7–9 report the findings for those who were not. The constant term of the former is 6.751, which is closer to the maximum value of the INDEX, 7,^10^ implying that the first subsample was compliant with contact avoidance almost to the maximum degree in advance. Then, we find that the gain-framed altruistic message promotes the contact-avoidance behavior in the short term in the second subsample of those who did not comply with such behavior before the experiment. This effect is small and statistically insignificant in column 4, while it is equal to 0.133 and statistically significant at the 5% level in column 7. The effect of the selfish message on neglecting the contact-avoidance behavior in the long term weakens in the two subsamples; however, the results suggest that it may be observed in the latter group. The selfish message effect is − 0.110 in column 9 (*p* < 0.10).

We reported in footnote 5 that the Cronbach’s coefficient alpha of the items for the INDEX was 0.6823, and then created three alternative indexes with higher alphas of 0.7094, 0.8188, and 0.9137, by excluding some items in the order of their low correlation with other items. We still find the same results shown in Table 9 in “Appendix” with the three indexes. The gain-framed altruistic message promotes contact-avoidance behavior in the first part of the experiment, and the selfish message decreases its compliance level in the second part. When using the full sample, the altruistic message’s effect for the second wave ranges from 0.072 to 0.078 (*p* < 0.05 for all), while the selfish message’s effect for the fourth wave ranges from − 0.076 to − 0.080 (*p* < 0.05 for all).

Overall, the study’s results indicate that when the Japanese government declared a state of emergency and people were required to refrain from going out and avoid contact with others, the gain-framed altruistic message promoted contact avoidance, particularly among people who went out more frequently before the experiment. To verify which specific behavior is promoted by this altruistic message, we decompose the contact-avoidance behavior INDEX and estimate the effect of the altruistic message on each item in the previously less compliant subsample. However, as mentioned above, some treatment effects may happen to be significant due to multiple outcomes. Thus, we consider the estimation results from this method only as reference information. The results in Table 3 show that those exposed to the gain-framed altruistic message may have reduced the frequency of going out to cafes and using public transportation, including trains and buses, compared to the control group.

**Table 3:** Effects on each item of contact-avoidance behavior INDEX

*Method:* fixed effects estimation	(1)	(2)	(3)	(4)	(5)	(6)	(7)	(8)	(9)	(10)
*Dependent variable:*	Bar	Restaurant	Cafe	Supermarket	Gym	Work	Public transportation	Plane	Small event	Large event
Each item of contact-avoidance behavior INDEX										
Wave2	0.020	0.166***	-0.032	0.249***	0.023	1.264***	0.381***	0.020	0.032	0.020
	(0.044)	(0.059)	(0.052)	(0.094)	(0.047)	(0.117)	(0.078)	(0.039)	(0.060)	(0.043)
Wave2 × Gain-framed altruistic	0.092	0.043	0.185**	-0.037	0.122*	0.301*	0.233**	0.122*	0.184*	0.089
	(0.071)	(0.087)	(0.080)	(0.130)	(0.071)	(0.169)	(0.113)	(0.063)	(0.097)	(0.066)
Wave2 × Loss-framed altruistic	0.058	− 0.105	0.072	0.100	0.061	-0.118	0.253**	0.064	0.053	0.093
	(0.061)	(0.079)	(0.074)	(0.132)	(0.070)	(0.172)	(0.117)	(0.053)	(0.079)	(0.059)
Wave2 × Selfish	0.015	− 0.118	0.023	-0.145	0.015	-0.181	0.033	0.041	0.043	0.025
	(0.058)	(0.076)	(0.070)	(0.127)	(0.065)	(0.158)	(0.109)	(0.052)	(0.082)	(0.059)
Wave2 × Selfish and altruistic	0.006	− 0.102	0.131*	-0.068	0.018	-0.056	0.028	0.009	0.088	0.033
	(0.066)	(0.085)	(0.074)	(0.135)	(0.068)	(0.166)	(0.114)	(0.060)	(0.091)	(0.065)
Wave2 × Simple	0.041	− 0.129	0.127*	-0.180	0.070	-0.024	-0.037	0.026	0.101	0.026
	(0.064)	(0.088)	(0.077)	(0.130)	(0.071)	(0.162)	(0.112)	(0.059)	(0.087)	(0.060)
Constant term	6.795***	6.478***	6.672***	3.711***	6.796***	2.666***	5.370***	6.826***	6.642***	6.811***
	(0.009)	(0.012)	(0.011)	(0.019)	(0.010)	(0.024)	(0.017)	(0.009)	(0.013)	(0.009)
Number of observations	4226	4226	4226	4226	4226	4226	4226	4226	4226	4226
Number of participants	2113	2113	2113	2113	2113	2113	2113	2113	2113	2113

*Notes* Cluster robust standard errors at individual level are in parentheses; ***p < 0.01, **p < 0.05, *p < 0.1. In this analysis, we only use the subsample those who were previously less compliant with contact-avoidance instructions

### Infection-prevention behavior INDEX

We report the results obtained when using the infection-prevention behavior INDEX as the dependent variable. First, we find that the gain-framed altruistic message decreases the level of the index, mainly from the first to the second wave. This effect is observed in those who were originally more compliant with contact avoidance and went out less frequently. Second, we find that the selfish message also decreases the level of infection-prevention behavior INDEX. As in the case of the altruistic message, this effect is observed in those who originally went out less frequently.

In columns 1–3 of Table 4, none of the wave coefficients are statistically significant. This result implies that the level of the infection-prevention behavior INDEX in the second, third, or fourth wave is, on average, indifferent to that of the first wave. In column 1, the coefficient on the gain-framed altruistic message is − 0.107 (*p* < 0.10), and that on the selfish message is − 0.129 (*p* < 0.05). Both coefficients are negative, suggesting that those exposed to these messages decrease their level of compliance with the infection-prevention behaviors from the first to the second wave. This effect of the selfish message is also observed in column 2, although its statistical significance becomes weakened (− 0.109, *p* < 0.10). Those exposed to the selfish message may have relatively low levels of infection-prevention behaviors not only during the second wave, under the state of emergency but also during the third wave, when the declaration was removed.

**Table 4:** Message effects on infection-prevention behavior INDEX

*Method:* fixed effects estimation	(1)	(2)	(3)	(4)	(5)	(6)	(7)	(8)	(9)
*Dependent variable:*	Full-sample			Sub-sample A: Those who were previously more compliant with contact-avoidance instructions			Sub-sample B: Those who were previously less compliant with contact-avoidance instructions		
Infection-prevention behavior INDEX									
Wave2	0.062			0.082			0.042		
	(0.044)			(0.059)			(0.066)		
Wave2 × Gain-framed altruistic	− 0.107*			− 0.197**			− 0.017		
	(0.063)			(0.091)			(0.086)		
Wave2 × Loss-framed altruistic	− 0.045			− 0.085			− 0.003		
	(0.062)			(0.086)			(0.090)		
Wave2 × Selfish	− 0.129**			− 0.167**			− 0.093		
	(0.059)			(0.082)			(0.084)		
Wave2 × Selfish and altruistic	− 0.055			− 0.054			− 0.057		
	(0.061)			(0.087)			(0.085)		
Wave2 × Simple	− 0.044			− 0.065			− 0.023		
	(0.060)			(0.081)			(0.089)		
Wave3		− 0.064			− 0.012			− 0.117*	
		(0.048)			(0.068)			(0.067)	
Wave3 × Gain-framed altruistic		− 0.084			− 0.155			− 0.011	
		(0.067)			(0.095)			(0.095)	
Wave3 × Loss-framed altruistic		0.062			0.042			0.081	
		(0.066)			(0.093)			(0.095)	
Wave3 × Selfish		-0.109*			− 0.108			− 0.105	
		(0.063)			(0.090)			(0.089)	
Wave3 × Selfish and altruistic		-0.044			0.014			− 0.101	
		(0.065)			(0.092)			(0.092)	
Wave3 × Simple		0.043			0.027			0.058	
		(0.065)			(0.093)			(0.092)	
Wave4			0.002			0.081			− 0.079
			(0.049)			(0.067)			(0.072)
Wave4 × Gain-framed altruistic			− 0.042			− 0.115			0.032
			(0.069)			(0.098)			(0.097)
Wave4 × Loss-framed altruistic			0.010			− 0.104			0.126
			(0.066)			(0.091)			(0.096)
Wave4 × Selfish			− 0.050			− 0.074			− 0.021
			(0.065)			(0.087)			(0.096)
Wave4 × Selfish and altruistic			− 0.053			− 0.059			− 0.046
			(0.067)			(0.094)			(0.096)
Wave4 × Simple			− 0.018			− 0.126			0.092
			(0.067)			(0.092)			(0.099)
Constant term	4.969***	4.969***	4.969***	5.045***	5.045***	5.045***	4.893***	4.893***	4.893***
	(0.009)	(0.009)	(0.009)	(0.013)	(0.013)	(0.013)	(0.012)	(0.013)	(0.013)
Number of observations	8482	8482	8482	4256	4256	4256	4226	4226	4226
Number of participants	4241	4241	4241	2128	2128	2128	2113	2113	2113

*Notes* Cluster robust standard errors at individual level are in parentheses; ***p < 0.01, **p < 0.05, *p < 0.1. We employ the contact-avoidance behavior INDEX in the first wave and divide the analysis sample into two subsamples: those who were relatively compliant with contact-avoidance instructions before the experiment (Sub-sample A: INDEX ≥ 6.5, *N* = 2128) and those who were not (Sub-sample B: INDEX < 6.5, *N* = 2113)

The results in columns 4–9 indicate that the above effects of the gain-framed altruistic and selfish messages are observed in those participants who were previously more compliant with the contact-avoidance behavior and went out less frequently before the experiment. In column 4, the coefficient on the altruistic message is − 0.197 (*p* < 0.05), and that on the selfish message is − 0.167 (*p* < 0.05).

Overall, under the state of emergency, the gain-framed altruistic and selfish messages decrease the level of infection-prevention behaviors of people who originally went out less frequently. Since the infection-prevention behavior INDEX consists of a variety of behavioral items, including wearing a mask, hand washing, shopping tips, sleeping, and eating, the implications vary depending on which items are affected by the messages. The results in Table 5 show that those exposed to the gain-framed altruistic message experience a worsening in the quality of their rest, sleep, and meals. Furthermore, they more often touch their face and go shopping with multiple family members or in crowded stores than those in the control group. In addition, those exposed to the selfish message use phone calls or video calls instead of face-to-face communication less often than those in the control group. The results imply that these messages may have a backfire or side effects when further compliance is required from those who have already been compliant.

**Table 5:** Effects on each item of infection-prevention behavior INDEX

*Method:* fixed effects estimation	(1)	(2)	(3)	(4)	(5)	(6)	(7)	(8)	(9)	(10)	(11)	(12)	(13)	(14)	(15)
*Dependent variable:*	Cough etiquette	Hand washing	Avoid touching face	Wear a mask when talking	Avoid shaking hands	Phone or video call	Avoid shopping with members	Cashless payment	Take-out or home delivery	Mail-order service	Waer a mask when going out	Stay home if you are ill	Stay home even if you are not ill	Rest and sleep	Nutrious foods
Each contact-avoidance behavior															
Wave2	0.076	-0.008	0.279**	0.192*	0.085	0.290**	0.079	− 0.056	0.166	− 0.169	0.054	0.087	− 0.045	0.093	0.110
	(0.129)	(0.083)	(0.110)	(0.113)	(0.136)	(0.146)	(0.114)	(0.114)	(0.167)	(0.150)	(0.100)	(0.140)	(0.089)	(0.083)	(0.086)
Wave2 × Gain-framed altruistic	− 0.210	-0.089	− 0.310**	− 0.114	− 0.171	− 0.243	− 0.455***	− 0.116	− 0.152	− 0.068	− 0.198	− 0.224	-0.050	− 0.268**	− 0.283**
	(0.194)	(0.121)	(0.157)	(0.169)	(0.200)	(0.211)	(0.174)	(0.165)	(0.237)	(0.216)	(0.139)	(0.199)	(0.138)	(0.121)	(0.131)
Wave2 × Loss-framed altruistic	− 0.323*	0.003	− 0.047	− 0.056	− 0.198	− 0.573***	− 0.141	0.107	− 0.016	0.421**	− 0.062	− 0.116	− 0.003	− 0.127	− 0.147
	(0.190)	(0.123)	(0.156)	(0.161)	(0.187)	(0.206)	(0.163)	(0.160)	(0.230)	(0.209)	(0.147)	(0.205)	(0.126)	(0.118)	(0.125)
Wave2 × Selfish	− 0.355*	0.008	− 0.236	− 0.211	− 0.210	− 0.410**	− 0.244	− 0.109	− 0.246	0.220	− 0.205	− 0.298	− 0.046	− 0.139	− 0.019
	(0.184)	(0.110)	(0.151)	(0.161)	(0.190)	(0.205)	(0.173)	(0.162)	(0.229)	(0.210)	(0.135)	(0.203)	(0.122)	(0.111)	(0.123)
Wave2 × Slfish and altruistic	− 0.304	− 0.106	− 0.001	0.010	0.050	− 0.340	− 0.234	0.091	0.120	0.353	− 0.036	− 0.055	− 0.087	− 0.175	− 0.092
	(0.192)	(0.114)	(0.155)	(0.163)	(0.197)	(0.216)	(0.180)	(0.163)	(0.249)	(0.218)	(0.138)	(0.213)	(0.125)	(0.118)	(0.124)
Wave2 × Simple	− 0.272	0.180	− 0.042	− 0.012	0.021	− 0.258	− 0.022	− 0.006	− 0.166	0.093	− 0.010	− 0.329*	− 0.034	− 0.164	0.048
	(0.183)	(0.124)	(0.146)	(0.157)	(0.187)	(0.205)	(0.163)	(0.155)	(0.232)	(0.216)	(0.142)	(0.193)	(0.117)	(0.116)	(0.123)
Constant term	5.173***	6.129***	4.139***	4.870***	5.566***	4.377***	5.212***	4.450***	3.683***	3.707***	6.033***	5.227***	6.261***	5.903***	4.946***
	(0.028)	(0.017)	(0.022)	(0.024)	(0.028)	(0.030)	(0.025)	(0.023)	(0.034)	(0.031)	(0.020)	(0.030)	(0.018)	(0.017)	(0.018)
Number of observations	4256	4256	4256	4256	4256	4256	4256	4256	4256	4256	4256	4256	4256	4256	4256
Number of participants	2128	2128	2128	2128	2128	2128	2128	2128	2128	2128	2128	2128	2128	2128	2128

*Notes* Cluster robust standard errors at individual level are in parentheses; ***p < 0.01, **p < 0.05, *p < 0.1. In this analysis, we only use the subsample those who were previously more compliant with contact-avoidance instructions

## Supplementary results

Previous studies have reported an intention-behavior gap in the effects of messages to encourage COVID-19 preventive behavior (Falco & Zaccagni, 2020).^11^ Although this study shows that only the gain-framed altruistic message promotes people’s behaviors of refraining from going out and avoiding contact with others, the other four messages may strengthen people’s behavioral intentions. In this section, we conduct estimations using intention instead of behavior.

We measure the effect of nudge-based messages on participants’ intentions to adopt a contact-avoidance behavior using the Ordinary Least Squares method. The model specification reads as follows:

2 $$y_{i} = \alpha + \beta \;{\text{Behavior}}_{i} + \gamma_{1} N1_{i} + \gamma_{2} N2_{i} + \gamma_{3} N3_{i} + \gamma_{4} N4_{i} + \gamma_{5} N5_{i} + X_{i} \delta + u_{i} .$$

The estimation uses cross-sectional data from the first, second, third, and fourth waves. The dependent variable ${y}_{i}$ is participant $i$’s contact-avoidance intention INDEX. α is a constant term that represents the level of the index in the control group for each wave. $\mathrm{Behavior}$ expresses the level of the pre-survey contact-avoidance behavior INDEX, which is ascertained in the first wave and not influenced by nudge-based messages. This variable controls for the previous behavior.^12^$N1-N5$ are the same as those in Eq. (1). ${\gamma }_{1}-{\gamma }_{5}$ are the treatment effects and represent the impact of each nudge-based message on the contact-avoidance intention INDEX. ${X}_{i}$ is a vector of participant $i$’s covariates, including age, gender, educational years, marital status, information related to annual household income, and response time; ${u}_{i}$ is a random error term.

The results in Table 6 show that the gain-framed altruistic and the loss-framed altruistic messages increase the contact-avoidance intention INDEX in those who originally went out more frequently. The coefficients on the gain-framed altruistic message are 0.193 in column 10 (*p* < 0.05), 0.181 in column 11 (*p* < 0.10), and 0.216 in column 12 (*p* < 0.05). The coefficients on the loss-framed altruistic message are 0.165 in column 11 (*p* < 0.10) and 0.284 in column 12 (*p* < 0.01).

**Table 6:** Message effects on contact-avoidance intention INDEX

*Method:* ordinary least squares	(1)	(2)	(3)	(4)	(5)	(6)	(7)	(8)	(9)	(10)	(11)	(12)
*Dependent variable:*	Full-sample				Sub-sample A: Those who were previously more compliant with contact-avoidance instructions				Sub-sample B: Those who were previously less compliant with contact-avoidance instructions			
Contact-avoidance intention INDEX	1st Wave	2nd Wave	3rd Wave	4th Wave	1st Wave	2nd Wave	3rd Wave	4th Wave	1st Wave	2nd Wave	3rd Wave	4th Wave
Gain-framed altruistic	0.091	0.093	0.079	0.096	0.053	− 0.005	− 0.025	− 0.022	0.125	0.193**	0.181*	0.216**
	(0.065)	(0.065)	(0.068)	(0.070)	(0.089)	(0.086)	(0.096)	(0.095)	(0.094)	(0.095)	(0.097)	(0.103)
Loss-framed altruistic	0.101	− 0.002	0.067	0.124*	0.043	− 0.086	− 0.033	− 0.043	0.146	0.079	0.165*	0.284***
	(0.064)	(0.066)	(0.068)	(0.071)	(0.088)	(0.091)	(0.096)	(0.097)	(0.092)	(0.096)	(0.097)	(0.102)
Selfish	0.096	0.037	0.077	0.082	0.167**	0.098	0.070	0.043	0.025	-0.020	0.083	0.120
	(0.063)	(0.064)	(0.066)	(0.068)	(0.084)	(0.086)	(0.095)	(0.092)	(0.092)	(0.095)	(0.093)	(0.100)
Selfish and altruistic	0.103	0.073	0.040	0.086	0.091	0.075	0.018	0.008	0.112	0.078	0.061	0.169*
	(0.063)	(0.066)	(0.069)	(0.069)	(0.087)	(0.086)	(0.097)	(0.094)	(0.092)	(0.100)	(0.098)	(0.102)
Simple	0.087	0.018	0.114*	0.094	0.051	-0.024	0.110	0.001	0.121	0.064	0.119	0.184*
	(0.064)	(0.065)	(0.067)	(0.069)	(0.088)	(0.089)	(0.093)	(0.091)	(0.092)	(0.095)	(0.098)	(0.104)
Contact-avoidance behavior INDEX before 1st Wave	0.255***	0.208***	0.191***	0.243***	0.213	0.275*	0.294*	0.409**	0.257***	0.201***	0.168***	0.227***
	(0.034)	(0.034)	(0.034)	(0.034)	(0.158)	(0.161)	(0.174)	(0.170)	(0.048)	(0.047)	(0.046)	(0.046)
Constant term	2.769***	2.920***	2.782***	2.632***	2.869***	2.137*	1.936	1.338	2.983***	3.358***	3.107***	2.943***
	(0.269)	(0.271)	(0.272)	(0.264)	(1.084)	(1.098)	(1.194)	(1.157)	(0.374)	(0.376)	(0.368)	(0.358)
Number of observations (participants)	4241	4241	4241	4241	2128	2128	2128	2128	2113	2113	2113	2113
R-squared	0.150	0.130	0.129	0.130	0.166	0.171	0.150	0.142	0.107	0.079	0.086	0.095

*Notes* Cluster robust standard errors at individual level are in parentheses; ***p < 0.01, **p < 0.05, *p < 0.1. We employ the contact-avoidance behavior INDEX in the first wave and divide the analysis sample into two subsamples: those who were relatively compliant with contact-avoidance instructions before the experiment (Sub-sample A: INDEX ≥ 6.5, *N* = 2128) and those who were not (Sub-sample B: INDEX < 6.5, *N* = 2113)

The above results imply that the two types of altruistic messages reinforce the immediate intentions even after repeatedly displayed, though not every time. However, the previous section showed that those messages do not promote compliance behavior in the third and fourth survey waves. There are two potential explanations. First, the findings might indicate the intention-behavior gap. Second, the results might be affected by differences in the timing of the intention and behavior assessments. As explained in Sect. 2, all survey waves captured participants’ behavioral intentions approximately one week after being exposed to the nudge-based messages. In addition, the second wave captured their behavior approximately 1 week after the first wave. In this period, the timing of the intention and behavior assessments coincides. On the other hand, the third and fourth waves captured participants’ behavior more than 1 month after being exposed to nudge-based messages. Therefore, in the second to the third or fourth wave, their behavior might have changed immediately after being exposed to the message: however, the above assessment method might have failed to capture the behavioral change. For instance, since the gain-framed altruistic message changes the behavior for 1 week from the first to the second wave, this possibility may be more promising in the message. On the contrary, since the loss-framed altruistic message has never shown any behavioral change effect throughout the experimental period, the results may be explained by the intention-behavior gap.

Unlike the two altruistic messages, the other three nudge-based messages do not keep strengthening the intention to avoid contact with others over time, even though some significant effects are observed in spots. For example, in column 5, the coefficient on the selfish message is 0.167 and statistically significant at the 5% level. This result implies that immediately after being exposed to this message in the first wave, those who originally went out less frequently strengthen their intention to avoid contact. However, we have shown that selfish messages do not promote contact-avoidance behavior from the first to the second survey. We even find a decrease in the level of the infection-prevention behavior INDEX. As mentioned above, the timing of the assessments of intention and behavior coincides during this period. Thus, we can state that the results for the selfish message support the intention-behavior gap. In other words, although the selfish message strengthens the contact-avoidance intention, it does not promote contact-avoidance behavior and may even worsen it.

## Discussion, limitations, and conclusions

We conducted a four-wave online survey experiment to examine how five types of nudge-based messages influence people’s self-reported behavior to prevent the spread of COVID-19. The results show that only a gain-framed altruistic message, emphasizing that behavioral adherence protects the lives of people close to the participants, reduces the frequency of going out and promotes contact avoidance.

The behavioral change induced by the gain-framed altruistic message is observed during the first part of the experiment when the Japanese government declared a state of emergency and the social need for people to refrain from going out was high. Concretely, people who viewed the message may have reduced the frequency of going out to cafes and using public transportation. This behavioral change disappears in the second part of the experiment when the state of emergency was removed. However, the intention to refrain from going out and avoid contact with others increases immediately after being exposed to this altruistic message, even during that period. In other words, the behavioral change effect of the gain-framed altruistic message could be short term, while its effect of changing perceptions and strengthening intentions could be long term, at least to some extent.

The behavioral change induced by the gain-framed altruistic message is also observed among those who went out more frequently and were relatively less compliant with contact avoidance before the experiment. The ceiling effect is one possible reason why the behavioral change effect is not observed in the other group. The contact-avoidance behavior INDEX of that group before the experiment was 6.751, which is close to its maximum value of 7. They may have had almost no room for further improvement. Barari et al. (2020) also use the ceiling effect, explaining that their nudge-based messages do not have an additional effect on those who are complying with preventive instruction.

The other four nudge-based messages do not encourage actions to prevent the spread of the infection. The loss-framed altruistic message, similar to the above message in the gain-frame, increases participants’ compliance intentions in the second part of the experiment. However, no behavioral change is observed at any point. The selfish message also increases the compliance intention in the first part of the experiment, while it actually worsens the infection-prevention behavior. As in Jordan et al. (2020), multiple messages can reinforce the intentions: however, only the gain-framed altruistic message is found from this study to have the behavioral change effect.

Here, we should note that the gain-framed altruistic message is also not a panacea. Although this message has the expected behavioral change effect on those who were relatively noncompliant with contact avoidance, it could have the adverse effects of decreasing the compliance level of infection-prevention behaviors on the remainder of the sample. For example, after being exposed to this altruistic message, the latter subgroup might have increased the frequency of shopping with multiple family members and in crowded stores and decreased the frequency of getting quality sleep and eating nutritious meals. The impact on sleep and eating, in particular, might be a side effect of requiring further compliance from those who already have been compliant. Another study suggests that the direction of the effect of interventions to prevent the spread of infection may be reversed in different groups (Krpan et al., 2021). The study shows that informational intervention further promotes contact avoidance in those who recently embraced avoidance, while reducing the frequency of contact avoidance in those who previously avoided contact.

This study’s policy implication is that, to promote contact-avoidance behavior, gain-framed altruistic messages should be shared in places and at times when people who go out more frequently and are not engaged in contact avoidance are more likely to be exposed to the messages. Although the behavioral change is only observed in the early stages of the experiment and is, then, short-term, complying intentions increase with each message exposure. Some influence on participants’ behaviors may be observed immediately after browsing, although this study cannot fully capture it. In light of these findings, it would be effective to put gain-framed altruistic messages on educational posters in the city or on apps often used by those who go out. These applications have already been implemented in various countries and may have been working as expected. However, the short-term effect of the gain-framed altruistic message can be explained also by habituation (Groves & Thompson, 1970; Thompson & Spencer, 1966). It refers to the phenomenon that when an intervention is provided repeatedly, responses to it gradually decline. The habituation likely occurs with neutral interventions without financial rewards or penalties. Outside of this experiment, Japanese central and local governments have frequently used gain-framed altruistic messages, which may have spurred the habituation of the message effect.^13^ It would be more effective to deliver this message to the target in a timely manner rather than posting it regularly.

The loss-framed altruistic message and selfish and altruistic message, both of which contain an altruistic component, do not have the same impact as the gain-framed altruistic message. This implies that the policymakers and practitioners need to carefully scrutinize the elements and wording of nudge-based messages, when using the messages as a countermeasure for COVID-19. Previous studies have reported that a loss-framed message is not always effective, while a gain-framed message promotes disease prevention behaviors (Detweiler et al., 1999; Toll et al., 2007). A more recent study on COVID-19 shows that preventive behaviors are promoted by both a threat-framed message, “hundreds of people will be lost,” and a gain-framed message, “we can save hundreds of lives.” However, the former generates more negative emotions (Heffner et al., 2020), which may inhibit behavioral changes. The result of the selfish and altruistic message may be explained by motivational crowding out, where referring to one’s gain blocks an increase in altruistic motivation (Gneezy & Rustichini, 2000). In addition, those exposed to selfish messages are likely to engage in behaviors with a high risk of infection, while the selfish and altruistic messages do not promote such actions. Emphasizing the interests of others over one’s interests might curb risky behaviors.

This study has some limitations. One is that there is a certain degree of response bias in our analysis sample, because we use a survey experiment with multiple follow-ups. Although we find no differences in psychological characteristics, including altruism and general trust, between the analysis sample and the remainder of the sample, the former contains a relatively large number of males, elderly people, and people with high annual household income. Such differences occur in the same way for each group. Therefore, this study may not fully capture the effects of nudge-based messages on young people. Another limitation is that there may be some differences between the effects of the messages on self-reported behaviors and actual behaviors (Hansen et al., 2021). For example, if we use GPS location data, we can determine the exact movement more accurately; however, this method can only capture its effect on movement. Using the survey experiment and the participants’ reports, we are able to examine the details of the behavior change and capture possible adverse and side effects of the altruistic message. Studies based on self-reported behaviors and those based on actual behaviors are complementary, and combining both data sources may enrich the interpretation of the results. Although this study has these limitations, it has sufficient academic and policy significance in terms of preventing the spread of COVID-19, because, to the best of our knowledge, no other study has conducted multiple surveys to examine the effects of nudge-based messages on the intention and behavior to prevent the spread of COVID-19 infection and their changes over time. Even after the start of vaccination, people will need to continuously avoid contact and prevent infection until herd immunity is acquired. We believe that this study’s findings will contribute to the realization of more effective use of nudge-based messages.

## Appendix

See Tables 7, 8, 9 and Figs. 4, 5, 6, 7, 8, 9, 10.

**Table 7:** Response rate for each wave

	Control	Gain-framed altruistic	Loss-framed altruistic	Selfish	Selfish and altruistic	Simple
2nd Wave	93.6%	93.3%	94.2%	92.3%	92.4%	94.2%
3rd Wave	86.8%	85.7%	86.1%	84.5%	84.9%	86.1%
4th Wave	79.3%	80.0%	78.9%	78.9%	78.8%	79.5%

*Note* We calculate out the response rates, setting the number of valid responses in the first wave as the denominator (888 in Control; 896 in Gain-framed altruistic; 883 in Loss-framed altruistic; 919 in Selfish; 878 in Selfish and altruistic; 898 in Simple)

**Table 8:** Checks for response bias

		Control		Gain-framed altruistic		Loss-framed altruistic		Selfish		Selfish and altruistic		Simple	
		Analysis	Other	Analysis	Other	Analysis	Other	Analysis	Other	Analysis	Other	Analysis	Other
	*N* =	704	184	717	179	697	186	725	194	684	184	714	184
Age	Mean	46.73	41.14	46.29	41.34	46.68	40.82	45.96	42.75	46.70	42.60	46.45	41.97
	S.D	[13.62]	[13.34]	[13.53]	[13.85]	[13.48]	[13.91]	[13.76]	[14.03]	[13.87]	[13.79]	[13.92]	[12.96]
Female dummy	Mean	0.49	0.60	0.50	0.59	0.50	0.53	0.50	0.61	0.52	0.51	0.49	0.58
	S.D	[0.50]	[0.49]	[0.50]	[0.49]	[0.50]	[0.50]	[0.50]	[0.49]	[0.50]	[0.50]	[0.50]	[0.49]
Educational year	Mean	14.58	14.47	14.60	14.36	14.67	14.48	14.63	14.39	14.66	14.32	14.73	14.40
	S.D	[2.09]	[2.13]	[2.05]	[2.03]	[1.97]	[2.05]	[2.03]	[2.06]	[2.06]	[2.08]	[2.06]	[1.95]
Married dummy	Mean	0.57	0.54	0.59	0.59	0.60	0.55	0.56	0.53	0.57	0.60	0.55	0.51
	S.D	[0.49]	[0.50]	[0.49]	[0.49]	[0.49]	[0.50]	[0.50]	[0.50]	[0.49]	[0.49]	[0.50]	[0.50]
Annual household income	Mean	606.16	598.98	622.92	600.92	611.07	578.99	631.46	547.06	627.84	633.72	628.98	555.86
(Unit: 10 thousand yen)	S.D	[344.90]	[369.76]	[378.02]	[358.47]	[359.44]	[286.01]	[373.06]	[324.75]	[343.79]	[306.14]	[376.24]	[308.08]
No income information dummy	Mean	0.19	0.24	0.16	0.17	0.19	0.19	0.19	0.15	0.19	0.22	0.17	0.20
	S.D	[0.39]	[0.43]	[0.37]	[0.37]	[0.39]	[0.39]	[0.39]	[0.36]	[0.39]	[0.42]	[0.38]	[0.40]
Altruism	Mean	2.85	2.88	2.88	2.84	2.92	3.02	2.83	2.97	2.92	2.86	2.93	2.91
	S.D	[0.99]	[0.98]	[1.01]	[1.01]	[1.01]	[0.95]	[1.03]	[1.05]	[1.01]	[1.05]	[1.00]	[0.97]
Trust	Mean	2.20	2.10	2.14	2.08	2.15	2.03	2.18	2.10	2.26	2.18	2.21	2.08
	S.D	[1.00]	[0.97]	[0.94]	[1.05]	[0.99]	[0.94]	[0.97]	[0.99]	[1.03]	[0.94]	[0.97]	[0.98]
Positive reciprocity	Mean	4.00	4.05	4.01	4.18	4.05	4.14	4.06	4.10	4.08	4.05	4.04	3.95
	S.D	[0.74]	[0.74]	[0.78]	[0.77]	[0.74]	[0.76]	[0.72]	[0.88]	[0.74]	[0.80]	[0.73]	[0.75]
Negative reciprocity (self)	Mean	3.10	3.05	3.04	3.08	3.12	3.13	3.05	2.97	3.04	3.13	3.03	3.05
	S.D	[0.99]	[1.00]	[0.98]	[1.10]	[0.97]	[1.03]	[1.00]	[1.17]	[1.04]	[1.09]	[1.01]	[1.11]
Negative reciprocity (other)	Mean	3.09	3.13	3.01	2.97	3.06	3.05	3.07	2.99	3.04	3.08	3.04	2.92
	S.D	[0.86]	[0.88]	[0.90]	[0.94]	[0.90]	[0.98]	[0.91]	[0.99]	[0.92]	[0.98]	[0.92]	[0.93]

*Notes* “Analysis” is the analysis sample of those who provided valid responses in all four survey waves, while “Other” is the other sample of those who did not. Altruism, Trust, Positive Reciprocity, Negative Reciprocity (self), and Negative Reciprocity (other) are the 5-point scale responses to the following questions: “I am willing to give to good causes without expecting anything in return (Altruism),” “I assume that people have only the best intentions (Trust),” “When someone does me a favor, I am willing to return it (Positive Reciprocity),” “I am willing to punish someone who treats you unfairly, even if there may be costs for you (Negative Reciprocity, self),” and “I am willing to punish someone who treats others unfairly, even if there may be costs for you (Negative Reciprocity, other)”

**Table 9:** Message effects on alternative contact-avoidance behavior INDEXES

*Method:* fixed effects estimation	(1)	(2)	(3)	(4)	(5)	(6)	(7)	(8)	(9)	(10)	(11)	(12)
*Dependent variable:*	Original INDEX			INDEX ver.2			INDEX ver.3			INDEX ver.4		
Contact-avoidance behavior INDEX	(The Cronbach’s coefficient alpha is 0.6823)			(The Cronbach’s coefficient alpha is 0.7094)			(The Cronbach’s coefficient alpha is 0.8188)			(The Cronbach’s coefficient alpha is 0.9137)		
	Full-sample	Sub-sample A	Sub-sample B	Full-sample	Sub-sample A	Sub-sample B	Full-sample	Sub-sample A	Sub-sample B	Full-sample	Sub-sample A	Sub-sample B
Wave2	0.057**	− 0.097***	0.214***	0.065***	− 0.077***	0.210***	0.006	− 0.066**	0.079**	− 0.016	− 0.067**	0.036
	(0.024)	(0.026)	(0.039)	(0.024)	(0.026)	(0.040)	(0.024)	(0.026)	(0.040)	(0.024)	(0.026)	(0.041)
Wave2 × Gain-framed altruistic	0.071**	0.007	0.133**	0.078**	0.001	0.152**	0.072**	0.010	0.134**	0.072**	0.024	0.120*
	(0.036)	(0.034)	(0.060)	(0.036)	(0.033)	(0.062)	(0.036)	(0.033)	(0.063)	(0.036)	(0.032)	(0.064)
Wave2 × Loss-framed altruistic	0.029	0.006	0.053	0.023	0.001	0.048	0.036	0.005	0.069	0.025	0.009	0.042
	(0.033)	(0.034)	(0.053)	(0.033)	(0.033)	(0.055)	(0.032)	(0.032)	(0.054)	(0.032)	(0.033)	(0.055)
Wave2 × Selfish	0.012	0.039	− 0.025	0.017	0.035	− 0.011	0.018	0.021	0.010	0.015	0.020	0.006
	(0.031)	(0.033)	(0.051)	(0.032)	(0.032)	(0.053)	(0.031)	(0.032)	(0.052)	(0.031)	(0.032)	(0.053)
Wave2 × Selfish and altruistic	0.033	0.054*	0.009	0.033	0.046	0.017	0.033	0.037	0.026	0.038	0.048*	0.026
	(0.033)	(0.029)	(0.057)	(0.033)	(0.028)	(0.059)	(0.033)	(0.027)	(0.059)	(0.033)	(0.027)	(0.060)
Wave2 × Simple	− 0.008	− 0.011	0.002	0.004	− 0.008	0.022	0.002	− 0.019	0.028	0.012	− 0.010	0.037
	(0.035)	(0.039)	(0.056)	(0.035)	(0.039)	(0.057)	(0.035)	(0.038)	(0.058)	(0.035)	(0.039)	(0.059)
Wave3	− 0.193***	− 0.282***	− 0.102***	− 0.188***	− 0.245***	− 0.130***	− 0.157***	− 0.169***	− 0.145***	− 0.131***	− 0.126***	− 0.135***
	(0.023)	(0.024)	(0.039)	(0.023)	(0.024)	(0.040)	(0.022)	(0.021)	(0.039)	(0.022)	(0.020)	(0.039)
Wave3 × Gain-framed altruistic	0.036	0.014	0.057	0.040	− 0.011	0.090	0.036	− 0.008	0.081	0.049	0.002	0.097
	(0.036)	(0.038)	(0.061)	(0.037)	(0.038)	(0.063)	(0.036)	(0.034)	(0.064)	(0.036)	(0.032)	(0.065)
Wave3 × Loss-framed altruistic	0.015	− 0.016	0.048	0.012	− 0.030	0.055	0.022	− 0.024	0.069	0.024	− 0.016	0.064
	(0.033)	(0.034)	(0.055)	(0.033)	(0.034)	(0.056)	(0.032)	(0.030)	(0.056)	(0.031)	(0.028)	(0.056)
Wave3 × Selfish	− 0.058*	− 0.031	− 0.091	− 0.056	− 0.046	− 0.071	− 0.063*	− 0.053	− 0.073	− 0.053	− 0.038	− 0.066
	(0.035)	(0.039)	(0.057)	(0.036)	(0.040)	(0.059)	(0.036)	(0.038)	(0.059)	(0.035)	(0.037)	(0.059)
Wave3 × Selfish and altruistic	0.030	− 0.005	0.064	0.030	− 0.010	0.069	0.041	− 0.014	0.096*	0.054	− 0.001	0.108*
	(0.034)	(0.039)	(0.056)	(0.035)	(0.039)	(0.057)	(0.033)	(0.035)	(0.056)	(0.033)	(0.033)	(0.058)
Wave3 × Simple	0.016	− 0.001	0.038	0.018	− 0.012	0.052	0.007	− 0.027	0.043	0.005	− 0.033	0.045
	(0.036)	(0.041)	(0.059)	(0.037)	(0.041)	(0.062)	(0.037)	(0.039)	(0.063)	(0.037)	(0.039)	(0.064)
Wave4	− 0.225***	− 0.346***	− 0.102**	− 0.230***	− 0.318***	− 0.140***	− 0.201***	− 0.235***	− 0.168***	− 0.165***	− 0.173***	− 0.157***
	(0.025)	(0.027)	(0.042)	(0.026)	(0.028)	(0.043)	(0.025)	(0.026)	(0.044)	(0.026)	(0.025)	(0.045)
Wave4 × Gain-framed altruistic	0.012	− 0.006	0.028	0.015	− 0.025	0.054	0.006	− 0.020	0.032	0.014	− 0.020	0.047
	(0.038)	(0.040)	(0.064)	(0.039)	(0.040)	(0.067)	(0.038)	(0.037)	(0.068)	(0.039)	(0.035)	(0.070)
Wave4 × Loss-framed altruistic	− 0.014	− 0.020	− 0.008	− 0.018	− 0.039	0.003	− 0.009	− 0.034	0.018	− 0.002	− 0.027	0.024
	(0.037)	(0.043)	(0.058)	(0.037)	(0.044)	(0.059)	(0.036)	(0.041)	(0.059)	(0.036)	(0.040)	(0.059)
Wave4 × Selfish	− 0.072*	− 0.041	− 0.110*	− 0.078**	− 0.063	− 0.099	− 0.080**	− 0.056	− 0.104*	− 0.076**	− 0.048	− 0.102
	(0.037)	(0.043)	(0.059)	(0.038)	(0.045)	(0.061)	(0.038)	(0.042)	(0.062)	(0.038)	(0.040)	(0.064)
Wave4 × Selfish and altruistic	0.009	0.004	0.011	0.003	− 0.003	0.008	0.010	0.001	0.018	0.020	0.008	0.031
	(0.037)	(0.041)	(0.061)	(0.038)	(0.041)	(0.063)	(0.037)	(0.038)	(0.063)	(0.037)	(0.037)	(0.064)
Wave4 × Simple	− 0.001	− 0.035	0.041	− 0.002	− 0.042	0.044	0.002	− 0.044	0.052	0.010	− 0.041	0.064
	(0.037)	(0.042)	(0.059)	(0.038)	(0.044)	(0.061)	(0.037)	(0.041)	(0.062)	(0.037)	(0.040)	(0.063)
Constant term	6.315***	6.751***	5.877***	6.529***	6.937***	6.117***	6.761***	6.971***	6.549***	6.851***	6.983***	6.717***
	(0.006)	(0.007)	(0.011)	(0.007)	(0.007)	(0.011)	(0.006)	(0.006)	(0.011)	(0.006)	(0.006)	(0.011)
Number of observations	8482	8482	8482	4256	4256	4256	4226	4226	4226	4226	4226	4226
Number of participants	4241	2128	2113	4241	2128	2113	4241	2128	2113	4241	2128	2113

*Notes* Cluster robust standard errors at individual level are in parentheses; ****p* < 0.01, ***p* < 0.05, **p* < 0.1. We employ the contact-avoidance behavior INDEX in the first wave and divide the analysis sample into two subsamples: those who were relatively compliant with contact-avoidance instructions before the experiment (Sub-sample A: INDEX ≥ 6.5, *N* = 2128) and those who were not (Sub-sample B: INDEX < 6.5, *N* = 2113)

## Footnotes

1 For example, BBC News reported that healthcare professionals in the UK shared a video on social media with the message of “If you choose to stay at home, you will save lives.” New Zealand Police tweeted that “We can save the human race by lying in front of the T.V. and doing nothing.”

2 In the first wave, we set a question to ascertain whether participants read our questionnaire and excluded from the follow-ups those who were found not to read it, further excluding from the analysis some participants who provided outlier answers (e.g., flying every day at a time before the experiment).

3 More precisely, we carry out the fourth wave when the number of confirmed new infections began to increase again.

4 “Avoiding the 3 Cs” is one of the slogans created by Japanese experts on countermeasures on COVID-19. Governments and media have frequently used this expression.

5 The Cronbach’s coefficient alpha for the responses in the first wave is 0.6823, which is lower than the general standard of 0.7 and thus raises concerns about their internal consistency. When we exclude some behavioral measures in the order of their low correlation with other measures, the alpha increases to 0.7094 (without the measure 4), 0.8188 (without the measures 4, 6), and 0.9137 (without the measures 4, 6, 7). In Sect. 3, we will use the indexes with more reliable alphas and confirm that our analysis results do not change.

6 The related study of Lunn et al. (2020) also uses a comprehensive index to evaluate the effectiveness of nudge-based messages.

7 The Cronbach’s coefficient alpha for the responses in the first wave is 0.8690, supporting that the responses have necessary internal consistency.

8 We confirm that there is no difference in the estimated results between when, respectively, using the two periods of data and the data of all the four periods simultaneously.

9 The related study of Krpan et al. (2021) also conducts the subgroup analysis based on pre-experimental compliance levels. Consequently, showing the possibility that the direction of the effects of nudge-based interventions to prevent the spread of infection is reversed across the subgroups.

10 However, the null hypothesis that the constant term is equal to 7 is statistically rejected at the 1% level.

11 The Intention-Behavior Gap has been reported in other topics, including commuter behavior and job search behavior (Abel et al., 2019; Kristal & Whillans, 2020).

12 As evaluated in the previous section, the nudge-based messages could influence the behavior, which is ascertained in the second, third, and fourth waves. Thus, we only control the pre-survey behavior also in the estimations with the data of the following wave.

13 In March 2020, the central government’s expert panel on the COVID-19 stated that if we simply avoid “3 Cs,” we can stop many people from becoming seriously ill and save lives. In April, they used the phrase “protecting the lives of those closest to us” in a document introducing tips for reducing contact with others by 80% (The Japanese government’s expert panel on the COVID-19, 2020a, b).
